# The Role of Family Time Together in Meeting the Recommendation for Physical Activity among Primary School Children

**DOI:** 10.3390/ijerph17113970

**Published:** 2020-06-03

**Authors:** Agata Korcz, Jana Krzysztoszek, Marlena Łopatka, Mateusz Ludwiczak, Paulina Górska, Michał Bronikowski

**Affiliations:** 1 Department of Didactics of Physical Activity, Poznan University of Physical Education, Królowej Jadwigi 27/39, 61-871 Poznań, Poland; krzysztoszek@awf.poznan.pl (J.K.); wyskok@awf.poznan.pl (M.Ł.); mateuszl92@o2.pl (M.L.); paula.g3@wp.pl (P.G.); bronikowski.michal@wp.pl (M.B.); 2 Mikołaj Kopernik School of Communications, Przełajowa 4, 61-622 Poznań, Poland; 3 Chair and Department of Bromatology, Poznan University of Medical Sciences, Marcelińska 42, 60-354 Poznań, Poland

**Keywords:** family time, physical activity, children, primary school

## Abstract

Family time may have an influence on children’s physical activity (PA) participation or may contribute to increased sedentary behaviour. The aim of this paper was to examine whether spending family time is associated with the PA of children aged 10–11. Cross-sectional data on 158 primary school children (80 girls and 78 boys) with a mean age of 10.6 ± 0.49 years were collected. Weight and height were used to calculate body mass index. The level of moderate-to-vigorous physical activity (MVPA) was determined with a physical activity screening measure. Participants wore a Vivofit^®^ wrist band activity tracker to measure their daily number of steps. The Health Behaviour in School-Aged Children questionnaire was used to obtain information from children about the frequency of activities undertaken by the family. Analyses entailed descriptive statistics of the total sample and by gender, *t*-test, and the Mann–Whitney U-test to examine the gender differences and Spearman’s correlation coefficients. It was found that 32.3% of the children did not accomplish at least 60 min per day on ≥5 of the seven days and more than 75.9% of them did not accumulate at least 12,000 or more steps daily. More boys than girls tended to be sufficiently active and met the guideline of at least 60 min per day on ≥5 of the seven days (70.5% and 65.0%, respectively) or 12,000 steps per day (25.8% and 10.0%, respectively). The number of steps during the weekday was significantly and inversely associated both in girls and in boys with active family time (*r* = −0.27 and *r* = −0.25, respectively), and with total family time (*r* = −0.28) and non-active family time (*r* = −0.25) only in boys. Average MVPA was also inversely correlated with active family time (*r* = −0.31), non-active family time (*r* = −0.24), and total family time (*r* = −0.29) in boys. The correlation effect size values ranged between small to medium significant differences for these measures. The findings underscore the need for community-based PA programmes designed for whole families to meet the recommended PA of children and also to develop and promote active leisure activities among families.

## 1. Introduction

Inactivity in children and youth seems to be a rising problem and a worrisome concern for modern public health and education specialists. According to the Global Matrix 3.0 report [1], the overall rate of PA of children and adolescents is low in the vast majority of the 49 countries that were assessed. In Poland, Mazur [2] says that only 24.2% of 11–15-year-olds met 1 h of MVPA per day. The age group 10–17-year-olds met the recommendation even less 21.5% [3]. Childhood and adolescence represent important life stages for primary prevention [4] and future behavioural patterns. Physical education (PE) during school among children has been widely recognized as a foundation for engagement in PA at adulthood. Indisputably, school PE is a significant component of daily PA [5]. However, the implementation of PE as the sole PA opportunity provides inadequate amounts of PA during the school day [6]. Therefore, PA opportunities should not be considered solely in relation to PE, but also before and after school, during school breaks and where possible [7]. It has to be highlighted that the foundations of PA behaviours are influenced by “different sectors” like schools, families, community, government, or even by media. In this paper, we would like to focus mainly on the family time aspect in the context of PA.

In the context of children’s PA level, parents are considered “guardians” [8], inhibiting or promoting a healthy lifestyle and activity of their children. Parental involvement in their children’s activity has both an immediate impact on a child’s current activity level and a lasting effect on subsequent activity during adulthood [9]. That is why more and more research is focused on the role of parents as the main factor in increasing the child’s PA. More specifically, parents may exert a significant social influence over their child’s PA through a variety of mechanisms which include parental encouragement, beliefs, and attitudes towards PA, by being active with their child, role modelling, involvement, and facilitation, such as logistic support and fee-paying [10,11,12]. According to Davison et al. [13], maternal and paternal involvement is qualitatively different, but both influence their child’s activity. Mothers have been found to provide more assistive support, promoting involvement through the reduction of barriers (e.g., payment of fees) and the provision of verbal encouragement. In contrast, fathers are characterised as fulfilling an ‘activation’ role in the development of their children, more overtly influencing their child’s activity, such as by planning family outings specifically to engage in activity and participating in activity directly with their child [13].

Only 20% of young people in Poland (12–18-year olds) declared being physically active with their fathers and 18.5% with their mothers [14], and 42.8% with their siblings [2]. It has been found that 51.6% of 12–18-year olds reported that parents always or almost always give support to their participation in PA materially (by providing resources), 64.5% consider themselves enabled to participate in PA, 41.4% get emotional support, 17.3% receive support in planning activities [14].

A literature review of studies examining the associations between parent and child PA behaviour deliver mixed and inconclusive results. Gustafson et al. [15], in their comprehensive 34-study review with respect to parent–child PA levels, stated that the results are ambiguous, and reported that associations of child activity with parent activity may, in fact, be mediated by differences in support and encouragement, not modelling. O’Connor et al. [10] in a systematic review claimed that one might have expected that engaging family members in a family PA or exercise programme with their children would be a promising strategy, but only two pilot studies had some effect on children’s PA and four had no effect or negative results.

Edwardson et al. [12] in a systematic review indicated that parental influence can be important for different types/intensities of PA in young people. In children (6–11 years) parents played an important role in MVPA, overall PA, and leisure-time PA. This was accomplished through direct involvement and being active role models. Additionally, parents were a part of organised PA through a combination of methods such as modelling, transport, and encouragement [12]. Meanwhile, Garriguet in “Health Reports” [16] indicated that parental role modelling and support have independent effects on a child’s (6–11 years) level of PA, as the effect amounted to an increase of 5–10 min in a child’s MVPA for every additional 20 min of the parent’s MVPA. Furthermore regardless of parents’ PA, supporting children through enrollment in lessons or league or team sports led to further increases in children’s PA. Girls who maintained MVPA had parents who reported sustained levels of logistic support across ages 9–15 years [10]. Other studies also show that providing logistical support is associated with increased PA [17,18,19], and therefore may be one of the most important sources of parental influence on children’s activity in this particular period of development. Other parenting strategies that may positively impact children’s PA include incorporating activity into family recreational routines, making activity-related equipment available at home, identifying safe places in the community that children can easily access, and finding activities to do outdoors for all weather conditions [17].

It is significant that PA in early childhood has been linked with an increased probability of engagement in PA in adolescence [20,21] as the habits of activity are formed early in our lifespan. Parents and the whole family especially can strongly influence children’s PA behaviours through role-modelling-parental direct help and creating opportunities to exercise in free time is associated with increased adolescent PA of the children [12].

At that early stage, where strong bonds with parents are influencing the child’s behaviours, thus setting the origins of their lifestyle, PA engagement of both fathers and mothers seems important. Given the fostering role in their children’s development within the family, the role of ‘physically active leader’ is often associated with fathers [22], with mothers being usually less active and having less influence on their children’s PA [23], but parenting styles of both parents are also independently associated with obesity risk in preschool children [24]. In a study by Bronikowski et al. [25], moderately positive changes were shown in physical fitness and PA parameters of both parents and their children involved in a 15-week intervention programme with PA twice weekly, but there were some other noted benefits such as more frequent family social behaviours (walks, meals, visiting relatives) and ‘do-together’ family leisure PA time. Moreover, in the research of Lam and McHale [26] and Badura et al. [27], it can be seen that parent–youth involvement (from middle childhood through adolescence) in joint physical activities was associated with more time spent in such activities by adolescents. It is important to emphasise in the context of studying the role of the family in the PA of children that parents have a bigger influence than PE teachers in promoting PA in adolescents, regardless of age, sex, and physical condition [28].

## 2. Purpose of Research

The aim of the study was to assess the association between spending family time together and PA among children aged 10–11.

## 3. Methods

### 3.1. Study Design and Participants

A cross-sectional study on a convenience sample of 4th-grade pupils aged 10-and 11-years-old (*N* = 158; 80 girls and 78 boys) was conducted in 2017/2018. The study involved three primary schools in a Polish city, where principals and parents with children agreed to take part in the study. The participants were recruited from the same grade level of standard urban schools. There was some data loss due to sickness of the children (participants) or technical problems with the wrist band activity tracker. The response set for children was 98.7%. Only a complete set of data was used for the statistical analyses. The questionnaire was completed in whole-class groups during one PE class in quiet conditions and took approximately 20 min to complete. Body mass and height data were collected by trained personnel with the use of anthropological instruments. Body height was measured to the nearest 0.5 cm using a portable stadiometer, and body mass was measured to the nearest 0.1 kg using electronic scales (Tanita Corporation, Tokyo, Japan). Body mass index (BMI), as a measure of body composition, was calculated as body mass/stature^2^ (kg/m^2^). Based on their BMI values, and participants were categorised into normal weight, overweight and obese groups according to age and sex-specific growth charts [29]. Anthropological measures were also taken during one PE class, taking about 20 min.

### 3.2. Physical Activity

Measuring PA was done subjectively (with a questionnaire) and objectively (with a wrist band activity tracker). The level of MVPA was determined with a physical activity screening measure [30]. Previous studies have shown acceptable reliability and validity of the MVPA measure [31,32]. Also, this measure was used earlier in Poland [25,33]. This measure corresponds to the average number of days per week with at least 60 min spent undertaking various forms of PA, during which, in the participants’ subjective opinion, their heart rates increased and they experienced a feeling of shortness of breath (higher breathing frequency). Participants were asked to answer two questions: over the past 7 days, on how many days were they physically active for a total of at least 60 min per day?; over a typical or usual week, on how many days were they physically active for a total of at least 60 min per day? The MVPA index was calculated based on the following formula: MVPA = (P1 + P2)/2, where P1 is the number of physically active days during the past 7 days and P2 is the number of physically active days during the typical (usual) week [30]. According to previously conducted studies [30,34], it may be accepted that children who were physically active for a total of at least 60 min/day on ≥5 of the 7 days met the PA guidelines. Therefore, in the present paper, time spent in MVPA was dichotomised as (1) MVPA ≥ 5 days/week (recommended level), or (2) MVPA < 5 days/week (below-recommended level).

Additionally, participants wore a Vivofit^®^ wrist band activity tracker (Garmin, Lenexa, Kansas, USA) 24-h a day for 8 consecutive days. The Vivofit^®^ provided high validity in measuring daily step counts [35]. During all walking conditions, participants were equipped with the following devices and carried them in standardised positions on the body. For all devices, participant data (sex, age, body height, and body weight) were entered for each participant before use. Tudor-Locke et al. [36] indicated that we can expect among children (typically 6–11 years), boys to average 12,000–16,000 steps/day and girls to average 10,000–13,000 steps/day. Meanwhile, Colley et al. [37] propose that 12,000 steps per day be used as a target to determine whether children and youth aged 6–19 are meeting the current PA guideline of 60 min of daily MVPA. Therefore, children were classified into two groups according to the mean value of steps taken during the day: (1) <12,000 steps/day was the group that did not achieve the PA recommendations; (2) ≥12,000 steps/day was the group that achieved PA recommendations.

### 3.3. Family Time Together

In order to measure family relationships and the frequency of activities undertaken by the family together, a selected question from the Health Behaviour in School-Aged Children (HBSC) questionnaire was used [31]. The list of things which some families do together includes 8 items: (1) watching TV or a video together, (2) playing indoor games together, (3) eating a meal together, (4) going for a walk together, (5) going places together, (6) visiting friends or relatives together, (7) playing sports together, and (8) sitting and talking about things together. Children were asked how often they had done any of these activities and spent time together in shared activities. Possible answers ranged from every day (coded as 5) to most of the days of the week (coded as 4), once a week (coded as 3), less than once a week (coded as 2), and never (coded as 1). The higher the mean values, the higher probability for the certain activity to be performed with the family. For the purpose of this article, we have created new variables: “total family time” (all 8 items), “active family time” (items: going for a walk, going places and playing sports) and “non-active family time” (itmes: watching TV, playing indoor games, eating a meal, visiting friends/relatives and sitting and talking), by adding the points score (1, 2, 3, 4, and 5 points for answers never, less than once a week, once a week, most of the days of the week, and every day, respectively). All new variables have been standardized as z-scores. The internal consistency for total family time scale was α = 0.75, for active family time scale was α = 0.70, and for non-active family time scale was α = 0.60.

### 3.4. Ethics

The research protocol was approved by the Ethics Committee of the Local Bioethics Committee of the Karol Marcinkowski University of Medical Sciences in Poznan (decision no. 947/16). Written informed consent was obtained from the schools and parents prior to data collection. Participants could withdraw from the study at any time without any explanation.

### 3.5. Statistical Analysis

The Statistical Package version 13.0 software package was used to conduct data analysis. The first analyses included descriptive statistics, primarily frequencies, provided understanding distributions of the respondents’ gender, PA and variables of family time spending together in shared activities. To compare the mean differences between the participant groups, the data were split on the basis of each category, and then a *t*-test was conducted to compare between groups for parametric data, Person’s χ^2^ for categorical variables and the Mann–Whitney U-test for non-parametric data. Spearman’s correlation coefficient was used to measure the strength of association between children’s family time spent together in shared activities and physical activity measures. The correlation effect size (*r*) was also calculated, with guideline values of correlation effect size 0.1, 0.3, and 0.5 regarded as small, medium, and large effects, respectively [38]. SPSS program has been used to create the standardized variables for total family time, active family time, and non-active family time. Statistical significance was set at *p* ≤ 0.05.

## 4. Results

The study sample consisted of 158 children (80 girls and 78 boys) with a mean (± standard deviation) age of 10.59 ± 0.492 years. Boys were older when compared with girls (10.68 ± 0.470 and 10.51 ± 0.503, respectively; *p* = 0.03). No significant difference was observed for body height, body mass and BMI mean values for boys and girls (Table 1). The prevalence of underweight, normal and overweight/obese are also summarised in Table 1. Out of 158 participants (50.6% girls and 49.4% boys), underweight was 15.2% and overweight, 15.2%. Furthermore, analysis by gender showed that a higher percentage of boys (19.2%) compared with girls (11.2%) were overweight. However, the percentage with normal weight was higher in girls (73.7%) than boys (65.4%).

Children’s descriptive characteristics, including average daily total PA and steps per day, are summarised in Table 2. On average, boys accumulated significantly greater MVPA, average steps per day and per weekday than girls (*p* = 0.09, *p* < 0.001 and *p* < 0.001, respectively). There was no significant difference in the mean number of steps during the weekend between the sexes. 32.3% of the children did not accomplish at least 60 min per day on ≥5 of the seven days and more than 77.2% of them did not accumulate at least 12,000 or more steps daily. Furthermore, more boys than girls tend to be sufficiently active and meeting the guideline of at least 60 min per day on ≥5 of the seven days (70.5 and 65.0%, respectively) or 12,000 steps per day (25.8% and 10.0%, respectively). However, those differences are not statistically significant. Significant differences were found between children classified in sufficiently and insufficiently active groups, both for the total group and for boys and girls in favour of those who are meeting guideline ≥60 min on ≥5 of the seven days (67.7% of the total group; 65.0% of girls; 70.5% of boys), and those who are not meeting the guideline ≥12,000 steps per day (75.9%; 90.0%; 64.1%, respectively).

The results presented in Table 3 show that eating a meal together or sitting and talking about things together are among the most frequent joint activities, while playing sports together and playing indoor games together are less frequent activities. In regard to gender, the same patterns in the analysed results/answers can be found across most of the family shared activities. No significant difference was observed in the mean values for boys and girls for family-shared activities.

Table 4 shows the Spearman correlation coefficients (*r*) between active family time, non-active family time, total family time and PA measures (average steps of weekday, average steps of weekend, average steps of 7 days and MVPA) by gender. Correlation coefficient values varied from −0.24 to −0.31 and they were all significant at the 0.05 error level. Steps during weekday was negatively correlated both in girls and boys with active family time (*r* = −0.27 and *r* = −0.25, respectively), and with total family time (*r* = −0.28) and non-active family time (*r* = −0.25) only in boys. Average MVPA was negatively correlated with active family time (*r* = −0.31), non-active family time (*r* = −0.24) and total family time (*r* = −0.29) in boys. The correlation effect size (*r*) values ranged between small to medium significant differences for these measures.

## 5. Discussion

The data presented in the current study complement the literature investigating the association between time spent in the family and PA of children aged 10–11. Our findings suggest that time spent in a family circle setting is not always time spent being physically active.

The results show significantly negative correlations both in girls and boys between steps during weekday and active family time active family time and with total family time and non-active family time only in boys. Average MVPA was negatively correlated with active family time, non-active family time time, and total family time in boys. However, those associations were in general between small to moderate. These results may indicate directions of changes in order to improve the quality of family leisure time in the context of PA.

According to Thompson et al. [39], despite the fact that most parents consider family involvement in PA to be important or very important for family life, there are also parents who place less emphasis on the value of being physically active as a family. They believe that the very fact of spending time together as a family without a significant understanding of the form or the way of spending it is crucial, whether it is PA or sedentary. However, in the MVPA range of the studied group, the results obtained were perhaps surprising in the context of other national [2,3] and international [1] reports, indicating a sufficient MVPA for most of children (67.7%) relative to the daily recommended dose of PA. Aubert et al. [1] in their international study provide evidence that children and adolescent populations in most countries that were assessed show a low prevalence of overall PA levels and indicate that the situation regarding the PA of children and youth is a universal concern worldwide. In the Polish cross-sectional studies around 75% of 11–15-year-olds do not meet 1 h of MVPA per day [2] and around almost 80% of 10–17-years-olds do not meet the same recommendation [3].

However, when compared objectively and subjectively measured PA, the obtained results were different in favour of the self-report PA data (MVPA measure). It was previously confirmed in a study by Kavanaugh et al. [40] that measuring MVPA via self-report versus accelerometry produces considerably different results in a sample of young adolescents. The reason for this might be that children (and adolescents) tend to overestimate their PA levels. Therefore, to obtain more accurate data, we should use well-validated, objective measures of MVPA and adopt apprortiate criteria. The reason for the discrepancy in subjectively and objectively measured PA data might be caused by a too-restrictive recommendation of daily number of steps per day (<12,000 steps/day was the group that did not achieve the PA recommendations and ≥12,000 steps/day was the group that achieved PA recommendations) for children in comparison to daily MVPA (MVPA ≥5 days/week-recommended level and MVPA <5 days/week-below-recommended level). Also, our results indicate that, overall, children have statistically significant greater PA levels on weekdays than on weekend days. The same results were obtained in a previous study conducted by Nilsen et al. [41].

Many children and adolescents do not meet PA guidelines and children’s PA levels decline steeply with age, particularly into adolescence [42,43,44]. The analysed studies show that boys are more active than girls of all ages [45,46,47,48,49,50], which also confirmed our results (boys took more steps than girls during the weekday as well as during seven days). It seems that even though children have opportunities to participate in PA during the school day, they are not able to reach the recommended activity time per day, and therefore they may not be receiving the improved health benefits. Consequently, we have to look for ways to increase opportunities for PA outside school and outside of traditional PE class (traditional PE understood as organised in/by a school system). An example of such research is a PA programme made popular by a school in Stirling, Scotland: the Daily Mile. The aim is that each day, during class time, pupils run or walk outside for 15 min (~1 mile) at a self-selected place. The findings show that, in primary school children, the daily mile intervention is effective at increasing levels of MVPA, reducing sedentary time, increasing physical fitness and improving body composition [48]. For MVPA, a relative increase of 9.1 min per day was observed. For sedentary time, there was a relative decrease of 18.2 min per day [51]. Italian researchers have come to similar conclusions while implementing the daily mile in primary schools. In the post-test period, the experimental group (*n* = 486 children) showed improvement in the 6-min run test and standing long jump results [52]. Another an innovative method for increasing PA in the school setting are brief bouts of PA during the school day. The programme, called “1 km a day”, consisted of performing a physically active break during the school day—walking or running 1 km outside the classroom in the schoolyard. Students and teachers reported a high level of satisfaction with the activity and low organisational demand. Hence, it was easily included in the school day routine among 11–14-year-old students [53].

Jago et al. [11], who explored the associations between the PA, sedentary, and TV viewing patterns of 10–11-year-old children and their parents, stated that there were no associations between the time that parents and children spend engaged in PA. Additionally, higher parental TV viewing was associated with higher child TV viewing among both boys and girls. A study conducted by Bagley et al. [54] shows that TV viewing can be influenced not only by parents’ behaviour, but also by having siblings. Both boys and girls with siblings spend less time on TV viewing. For girls, it is also important whether they are brought up in a family with one or both parents. Girls from single-parent families spend more time on TV viewing [54]. This line of research has indicated that providing structure and guidance, such as setting limits on how much time a child can spend using screen media or the placement of screen media devices in the home are associated with lower screen media use [55].

Jago et al. [11] imply that the absence of an association between parent and child PA could be a function of the child’s age, with 10–11 years of age being a period when children’s cognitive decision-making abilities increase and they begin to assert a degree of independence from their parents. The ages of 10–11 years old is a crucial period of change [48] as they move from childhood to adolescence and many seek opportunities for increased autonomy. Jago et al. [11] suggest that parental influence on the PA of 10–11-year-olds is likely to be facilitative in nature and not by modelling or copying of behaviours. Collectively, the parent and child data reinforce the view that 10–11 years of age is a period when parental influence on activity becomes less pronounced and the influence of friends increases, suggesting that dual-parent and friend approaches to increasing PA may be particularly effective at this age [56]. When considering the influence of parents, it is worth considering that the influence of the mother may be different from that of the father. A study by Cleland et al. [57] shows that the mother has a greater impact on PA undertaken by younger boys (5–6 years) after school and during the weekend. Her role is also of greater importance in undertaking PA after school by younger girls (5–6 years old). In turn, in the group of older boys (10–12 years), the father has a greater impact on undertaking PA during the weekend [57]. In spite of it all, regarding the role of parenting in children’s PA behaviours, evidence suggests that more permissive approaches are associated with greater child engagement in MVPA [55,58]. In the analysed context, the time segments in which the joint activity of parents with children take place are also important. Fuemmeler et al. [59] claimed that greater parental MVPA was associated with increased child MVPA and were significantly correlated for many of the observed time segments (weekend, weekday, and weekday 3–7 pm). However, they established, that children of two highly active parents engaged in more MVPA than children of parents who engaged in very little MVPA. This study did not determine whether parental modelling, support, shared activities, or combinations of such factors were responsible for the parent-child activity aggregation found, or confirm the report of Jago et al. [11], that parents’ sedentary activity was significantly correlated with their children’s sedentary activity, but what was important proved that the MVPA levels of parents were predictive of children’s activity after school.

Considering our findings that parents spend little time with their children on joint PA and their non-compliance with MVPA recommendations, with an ambiguous picture of the importance and impact of modelling behaviour as a predictor of PA in children, it is worth undertaking further research in this area and seeking other options to increase children’s PA for future health. Tate et al. [60] suggest that parents who are less active may potentially soften/lighten negative effects of their relative inactivity by strategically encouraging their children’s own PA. They proved for less active parents, more encouragement for PA was associated with higher children’s MVPA. One of the primary mechanisms by which parents influence activity levels is through the provision of social support [61]. In the context of watching TV, developing strategies to change the home TV environment may be important for reducing children’s TV viewing. Also, Wilk et al. [62] indicate the effect of parental PA on child’s PA was not statistically significant, however, parental support for PA had a statistically significant indirect effect on child PA levels in girls and boys. The study of Welk et al. [63] examined the utility of a model to explain parental influence on children’s PA (children from elementary schools). Correlations between parent and child levels of activity were low, but children of active parents had higher scores on the parental influence measures and psychosocial correlates than inactive parents. The study of Welk et al. [63] provides further confirmation of the important influence that parents exert on their child’s PA behaviour. The study of Sigmundová et al. [64] indicated that the children of fathers and mothers who met the weekend recommendation of 10,000 steps were more likely to achieve the international weekend recommendation than the children of less active parents. Furthermore, the children of mothers who reached the weekday pedometer-based step count recommendation were more likely to fulfil the step count recommendation on weekdays than the children of less active mother [64]. Above all, if parents do not undertake PA themselves or try to avoid PA, they will not be able to convince children to be physically active and try to increase their PA.

Our study also describes the family time in joint activities. Both girls and boys spend time together with their families in a similar way. Eating a meal together and sitting and talking about things together are among the most frequent joint activities, while playing sports together and playing indoor games together are the less frequent activities, what was confirmed also in the study of Zaborskis et al. [65]. In the study of Vokacova et al. [66] reported time trends in joint family activities between 2002 and 2014. Compared with 2002, there was a slight increase in four out of the six selected joint family activities (watching TV or a video, playing indoor games, eating meals, going for a walk, going places, visiting friends or relatives, playing sports, sitting and chatting). In particular, the likelihood of engaging in joint active activities (sports and walks) increased in the 2002–2014 period. Conversely, adolescents watched TV with their parents less frequently. Moreover, families did not eat together as often as in 2002, which might have negative consequences for healthy adolescent development. Also, adolescents aged 11 got involved in joint family activities more than their older counterparts [66].

To sum up, family time seems to be important in terms of role-modelling PA, providing a safe and interesting backyard for children to play in, setting rules about how small screen entertainment is used at home, and providing healthy lifestyle. It is crucial to create and implement specific strategies/actions to increase children’s PA also beyond the school setting and traditional PE. The initiatives should include a multifaceted approach whereby children are provided increased opportunities for PA to achieve the recommended amount of daily PA. Parents are one of the primary providers of opportunities to be active, and therefore, have the possibility to increase the activity levels of their children. Strategies for increased parental support may include, for example, letters mailed home to each of the families, a school newsletter or/and social media.

The major strength of the study is the incorporation of well-validated, objective, and subjective PA measures on the same children (MVPA and number of steps). The two methods provide valuable information, as they both provide a picture of a habitual PA level. Yet, despite the study’s strengths, there are numerous factors that can affect the behaviours of children and youth [67], which we did not consider. As in any paper, the results of our study should be considered with respect to the limitations of the study. First, the data used in this study were cross-sectional and the study sample was not representative, because sample sizes were relatively small, especially when grouped by distinct sex and those who achieve and do not achive PA recommendations. Therefore, generalisability to other populations may not be appropriate. Second, as the data for family time together and PA (MVPA screening measure) were self-reported, their accuracy should be considered with caution. All analyses were univariable and did not take into account potential confounding variables such as socioeconomic status. Another limitation of the study is the lack of data concerning waist-to-hip ratio (WHR), we have based only on BMI measures (where BMI is a measure of heaviness, not fatness). Further research is needed in order to assess the associations between family time together and health behaviour in children.

## 6. Conclusions

The key finding of this study was that many potentially modifiable aspects of the family time together were negatively associated with primary school children’s PA. Our findings suggest that time spent in a family circle setting is not always time spent being physically active, and both girls and boys spend time together with their families in a similar way. Eating a meal together or sitting and talking about things together are among the most frequent joint activities while playing sports together and playing indoor games together are the less frequent activities. Results indicated that steps taken during weekday was negatively associated with active family time both in girls and boys, and with non-active family time and total family time only in boys. Average MVPA was negatively correlated with active family time, non-active family time, and total family time in boys. Also, the current study contributes to the literature by highlighting the importance of using well-validated, objective, and subjective measures of PA and a proper selection of criteria concerning the daily MVPA level and daily number of steps.

## Figures and Tables

**Table 1:** Descriptive statistics (mean, standard deviation and frequency) of the anthropometric indicators/measurements of the children by gender.

Variables	Total (*N* = 158)	Girls (*n* = 80)	Boys (*n* = 78)	*p*-Value
Age (years)	10.6 ± 0.49	10.5 ± 0.50	10.7 ± 0.47	**0.03**
Body high (cm)	144.1 ± 0.08	143.8 ± 0.08	144.4 ± 0.08	0.68
Body mass (kg)	37.0 ± 8.00	36.9 ± 7.99	37.1 ± 8.06	0.87
BMI (kg/m^2^)	17.6 ± 2.4	17.7 ± 2.47	17.6 ± 2.45	0.87
BMI categories (%)				
Underweight	24 (15.2%)	12 (15.0%)	12 (15.4%)	0.36
Normal weight	110 (69.6%)	59 (73.7%)	51 (65.4%)	
Overweight	24 (15.2%)	9 (11.2%)	15 (19.2%)	

Notes. BMI = Body Mass Index. *p*-values were derived from the t-test and Person’s χ^2^ for categorical variables (BMI categories). The significant analyses (*p* < 0.05) may be found as bold.

**Table 2:** Descriptive statistics (mean, standard deviation and frequency) of PA of the participants.

Variables	Total (*N* = 158)	Girls (*n* = 80)	Boys (*n* = 78)	*p*-Value
MVPA (d/w)	5.013 ± 1.40	4.825 ± 1.42	5.205 ± 1.37	0.09
≥60 min (≥5 d/w)	5.8 ± 0.75 (67.7%)	5.6 ± 0.68 (65.0%)	5.9 ± 0.79 (70.5%)	0.06
<60 min (<5 d/w)	3.37 ± 0.96 (32.3%)	3.3 ± 1.10 (35.0%)	3.5 ± 0.76 (29.5%)	0.48
*p*-value	**<0.001**	**<0.001**	**<0.001**	
Steps Weekday (s/d)				
	11.521 ± 3.03	10.724 ± 2.43	12.339 ± 3.37	**<0.001**
Weekend (s/d)	8.063 ± 3.43	7.763 ± 2.87	8.370 ± 3.92	0.27
*p*-value	**<0.001**	**<0.001**	**<0.001**	
7 days (s/d)	10.115 ± 2.80	9.400,0 ± 2.28	10.848 ± 3.10	**<0.001**
≥12.000 (s/d)	14.015 ± 1.58 (24.1%)	13.233 ± 1.58 (10.0%)	14.123 ± 1.61 (25.8%)	0.14
<12.000 (s/d)	8.964 ± 1.90 (75.9%)	8.852 ± 1.78 (90.0%)	9.013 ± 2.04 (64.1%)	0.64
*p*-value	**<0.001**	**<0.001**	**<0.001**	

Notes. PA—physical activity; d/w = days per week; s/d = steps per day. Sufficiently Active-meeting guideline at least 60 min of daily moderate- to vigorous-intensity physical activity (MVPA) (≥5 day/week) or 12,000 steps per day. *p*-values were derived from the t-test (to compare girls with boys) and from the Mann-Whitney U-test (to compare sufficiently with insufficiently active children). The significant analyses (*p* < 0.05 and *p* < 0.001) may be found as bold.

**Table 3:** Means and standard deviations of responses to questions on family time spent together in shared activities.

Shared Activity	Total (*N* = 158)	Percentage of the Answers “Every Day”	Girls (*n* = 80)	Boys (*n* = 78)	*p*-Value
Watching TV or a video together (pts)	3.36 ± 0.883	10.7	3.34 ± 0.940	3.38 ± 0.825	0.74
Playing indoor games together (pts)	2.81 ± 1.054	6.3	2.77 ± 0.993	2.85 ± 1.117	0.67
Eating a meal together (pts)	4.15 ± 0.991	44.3	4.09 ± 1.033	4.22 ± 0.949	0.41
Going for a walk together (pts)	2.94 ± 1.113	9.0	2.85 ± 1.115	3.04 ± 1.110	0.29
Going places together (pts)	3.08 ± 1.022	10.8	3.07 ± 1.028	3.10 ± 1.022	0.93
Visiting friends or relatives together (pts)	3.16 ± 1.064	12.02	3.20±1.152	3.13 ± 0.972	0.67
Playing sports together (pts)	2.78 ± 1.175	6.3	2.70 ± 1.152	2.87 ± 1.199	0.36
Sitting and talking about things together (pts)	4.21 ± 1.035	50.6	4.31 ± 1.038	4.10 ± 1.027	0.20

Notes. pts = points. Response codes: every day = 5, most days = 4, about once a week = 3, less often = 2, never = 1. *p*-values were derived from the *t*-test.

**Table 4:** The Spearman correlation coefficients (*r*) between variables: active family time, non-active family time, total family time and weekday, steps during the weekend and MVPA in children.

**Variables**	**Active Family Time**	**Non-Active Family Time**	**Total Family Time**
	**Girls**		
Average MVPA	0.13	−0.03	0.05
Steps 7 days	−0.16	−0.15	−0.16
Steps weekday	**−0.27**	−0.14	−0.22
Steps weekend	0.03	−0.03	0.01
	**Active Family Time**	**Non-Active Family Time**	**Total Family Time**
	**Boys**		
Steps weekend	**−0.31**	**−0.24**	**−0.29**
Steps 7 days	−0.17	−0.15	−0.17
Steps weekday	**−0.25**	**−0.25**	**−0.28**
Steps weekend	0.10	0.12	0.12

Notes. Significant correlations are indicated in bold. Correlation is significant at the *p* < 0.05 level.
